# Nursing Education in Saudi Arabia: History and Development

**DOI:** 10.7759/cureus.7874

**Published:** 2020-04-28

**Authors:** Khalid Abdullah S Aljohani

**Affiliations:** 1 Community Health, Nursing College-Taibah University, Medina, SAU

**Keywords:** nursing history, saudi arabia, nursing education, bachelor, postgraduate, development

## Abstract

Nursing education in Saudi Arabia commenced in 1948 with the introduction of a nursing diploma program for males. Numerous bodies have worked as nursing education providers since then, leading to an unsteady growth of the field due to the lack of a unified curriculum coupled with an absence of regulating bodies. The absence of nursing leaders and the lack of reliable nursing workforce data during the past few decades have contributed to a lack of maturity and growth of the Saudi nursing landscape compared to the international nursing trends. This review explores the history of nursing education and its development within the context of Saudi Arabia.

## Introduction and background

Since 1948, nursing in the Kingdom of Saudi Arabia (KSA) has gone through several transformations, and the profession is still undergoing a stage of development. The latest published statistics by the Ministry of Health (MOH) in KSA show that nurses represent 41% (83,596) of the MOH workforce. Specifically, Saudi citizens represent 63% of the entire MOH nursing workforce [1]. Furthermore, based on the current student capacity of nursing colleges, the projected number of nursing graduates during 2019-2027 is about 26,200 [2]. Therefore, to reach the recommended ratio of one nurse for every 200 Saudi citizens, KSA must recruit 185,722 expatriate nurses [2]. The recruitment of expatriate nurses throws up several challenges, including a competitive market, high turnover, financial costs, healthcare instability, and concerns regarding patient safety [3]. Therefore, establishing and enhancing domestic nursing education programs to meet the needs of the nursing workforce will be a key strategic requirement for the Saudi government.

Currently, there are 39 nursing programs across KSA. The nursing departments within allied health colleges operate many of these nursing programs [4]. The new trend is to separate these nursing departments from the host institutions to become independent colleges. As of now, nurses in KSA are classified into three categories according to the Regulations of Nurses Classification Article 17 issued by the Saudi Commission for Health Specialties (SCFHS). Since the governmental regulation to close nurse technician schools has come into effect, the nurse technician classification and registration are no longer utilized. Currently, the first category among the three classifications is a nurse specialist who holds a bachelor’s degree in nursing or equivalent from a recognized educational body in the country of issuance. The second category is a senior nurse specialist who has a master’s degree in nursing with a minimum of two years of clinical experience. The third category is a nurse consultant who has a Ph.D. in nursing with a minimum of three years of clinical experience after securing the Ph.D. [5]. Nursing registration data show that 46 nurse consultants (40% of which are Saudi citizens), 620 senior specialists (49% Saudi citizens), and 13,862 Saudi registered nurses with bachelor’s degrees in nursing are currently registered to be employed in KSA [2].

A search of the literature revealed limited sources in KSA to retrieve adequate information about the historical background of nursing education and the countrywide development of nursing as a profession. Moreover, there is a scarcity of published articles regarding specific nursing issues and trends in KSA, such as female nursing education and its contributions to the healthcare workforce and development of nursing education programs and future plans for nursing education in the country [6-9]. In addition, there is a lack of knowledge about the overall history and development of nursing education, from its beginnings at the Arabian-American Oil Company in 1948 until now. Therefore, this paper aims to provide a brief survey of the history and development of nursing education within the context of KSA, including details of nursing regulations stakeholders and their role in such development.

## Review

Methods

The study utilized the integrative literature review approach for utilizing sources to the maximum extent possible. The integrative framework was developed by Whittemore and Knafl and is composed of five phases: problem identification, literature search, data evaluation, data analysis, and data presentation [10]. The study followed the Preferred Reporting Items for Systematic Reviews and Meta-analysis (PRISMA) framework to enhance the reporting and presentation of data [11]. Bibliographic databases such as PubMed, Web of Science, EBSCO-CINAHL, Ovid, and Google Scholar were searched for the following keywords: “nursing, education, and Saudi Arabia.” The inclusion criteria for utilizing the published literature were as follows: peer-reviewed articles in English and a focus on nursing education in KSA. Governmental sources including those in Arabic were included. Discussion articles, letters to the editor, and articles with weak documented references were excluded. Direct databases search revealed 222 articles while additional sources including governmental reports revealed 46 pieces. The removal of duplications resulted in a tally of 232 articles. The initial screening and full-text assessment revealed 31 related sources. Data evaluation was guided by our research objective about the history and development of nursing education in KSA. The search continued throughout the duration of the writing of this paper (February-April 2019).

Results

A Historical Overview of Saudi Nursing Education

Nursing education in KSA was initiated by parties/authorities other than the Ministry of Education (MoE). This might be attributed to the fact that technical education was outside the scope/mandate of the MoE at the time. An American male nurse who was working for the Arabian-American Oil Company in 1948 initiated the first documented nursing education classes [12]. Following the establishment of nursing as a profession in the MOH in 1954, the Saudi government launched the first official nursing school in 1958, enrolling 15 male students with elementary school education in the first nursing program of one-year duration [13]. The program duration increased to two years in 1961 [14]. In general, there were two nursing program pathways dominating nursing education at the time. The first trend began in 1958 when the government departments such as the MOH and Ministry of Defense were in need of nurses to employ and run their health organizations [8,15]. These programs recruited male and female students who had completed six years of elementary school [8]. The admission requirement was upgraded to intermediate level (ninth grade) in 1981 and to secondary school in 1992 [13,16]. The second trend was set in motion when the MoE was established in 1975 and started university level programs [17]. All enrolled students in these programs were female [18]. The acceptance of male students to the program started in 2004. Bridging to a bachelor’s program in nursing was offered to those who had graduated in professional programs, such as a nursing diploma or associate’s degree, to complete the Bachelor of Science in Nursing (BSN) degree. In the 1980s, the first Master of Science in Nursing (MSN) program for female nurses was introduced at King Saud University in Riyadh. The same program was introduced by the same university for male nurses in 2013. In July 2011, a royal decree ordered the transfer of the governance of 39 health institutes and colleges from the MOH to MoE. These institutes were merged into 15 governmental universities. Since then, all students have graduated with a BSN degree according to the universities’ system of running these programs. However, vocational nursing programs were reintroduced by the SCFHS in 2018. The introduction of these training programs has encouraged public and private healthcare organizations to take part in the programs’ operations as training centers and fulfill their nursing workforce needs without the need for university graduates.

Nursing Schools in Saudi Arabia

Based on the MoE statistics in 2017, of the 39 nursing colleges in KSA, 13 colleges have been established and run by the private sector. The rest of the colleges are operated by the government [2]. A total of 17,085 BSN students are enrolled in these various programs at Saudi higher education institutions [2]. Female students (76%, n = 13,001) represent the majority of BSN students in KSA, as well as in many other countries worldwide. The Saudi government provides international scholarships for Saudi citizens to study nursing. About 813 Saudi students are enrolled in different nursing schools in various foreign countries, mainly the USA and Australia [2]. These international colleges provide nursing education programs at different levels.

Bachelor’s Programs in Nursing in Saudi Arabia

According to the nursing education regulations in KSA, bachelor’s programs in nursing follow two pathways: the regular nursing program (RNP), and the bridging nursing program (BNP). Since its establishment, RNP has been designed as four years of academic study, followed by one year of internship [19]. On the other hand, the BNP is a two-year program, followed by six months of internship, for students who are already registered nurses with a diploma certificate. The National Commission for Academic Accreditation and Assessment (NCAAA), which was founded in 2004, monitors bachelor’s programs under the MoE governance. The first year of the RNP, which includes two semesters, focuses on the scientific pathway. Students learn general science courses, the English language, and communication and learning skills. These courses are essential requirements for a bachelor’s degree in nursing. General nursing science courses are introduced in the second and third years, while specialized nursing courses, such as critical and emergency nursing care and geriatric nursing care, are offered in the fourth year. The internship is organized and monitored by the university, while internship sites provide the internship training. Out of the 52 weeks of training, the internship includes four weeks of holidays, which are scheduled by the interns in coordination with the clinical placement authorities. Nursing interns undertake their rotations in several nursing departments, including emergency, intensive care, medical-surgical, pediatric, maternity (for female students only), and psychiatric, as well as primary health care clinics. Maternity nursing is delivered through two courses to male and female students. Unfortunately, male students do not have the opportunity to be exposed to real practice in perinatal care settings. However, they get extensive training on high-fidelity simulation manikins and equipment during their undergraduate education to compensate for the lack of such training. Generally, the internships at most Saudi universities utilize the supervised practice internship model in which a staff nurse supervises the student while the academic member works in liaison to ensure the smooth achievement of the internship plan. Students are required to pass the Saudi Nursing Licensing exam during or after their internship in order to be eligible to practice as a registered nurse upon the completion of the internship program.

Postgraduate Nursing Education

According to the SCFHS registry in 2017, there are 956 Saudi Arabian postgraduate nursing students. Among those, 365 are studying at national universities while the rest are on international scholarships [2]. The first master’s program in nursing in KSA was established by King Saud University in 1987, which enrolled only female students [20]. By 2013, male nurses were able to enroll in the master’s program at the same university. Currently, there are numerous master’s programs in nursing in government-run universities. These programs are of two years’ duration, provided as a full-time course only. Usually, the first semester includes general courses for different specialty programs in nursing, wherein the students learn about nursing theories, advanced learning and education skills, and nursing research and biostatistics. The ratio of theoretical to clinical components of the nursing courses in these programs varies among the specialties in master’s programs, with accumulative 42 credit hours, according to the Unified Law Organizing the Graduate Studies in Saudi Universities [21]. Universities offer the main nursing specialties, which include community health, psychiatric and mental health, nursing administration and education, medical-surgical nursing, and maternal-child nursing [22,23]. The only Ph.D. program in nursing in the country is offered by King Saud University, which was introduced in the academic year of 2019 [22]. In addition, a new initiative of providing a Doctorate in Nursing Practice (DNP) has been instituted by a collaboration between Saudi Aramco and Johns Hopkins University.

The Ministry of Education

As mentioned earlier, the Ministry of Higher Education has been merged into the MoE. The MoE is a governmental body responsible for approving and supervising higher education organizations, both in government and the private sector. Constituting the Deans of Nursing Colleges Committee (DNCC) was first suggested in February 2014 by one of the recommendations of the National Guard Health Sciences Conference. This suggestion was approved by the MoE and came into effect in November 2014, when the first meeting was held at King Saud Bin Abdulaziz University for Health Sciences in Jeddah (Minutes: Alfozan H, Jawharji I, Omar T, et al. Meeting One for the Deans of Nursing Colleges, 2014). The key role of the DNCC is to coordinate and support nursing programs as well as to work as a consultative body for the MoE in matters related to nursing issues.

Saudi Arabia Qualifications Framework

According to the recommendations of the Saudi Vision 2030, there is a need to enhance the education system with a focus on regulations and policies [24]. Therefore, a royal decree in October 2018 replaced the Education Evaluation Commission with the Education and Training Evaluation Commission (ETEC). There are four divisions under the ETEC, two of which are concerned with nursing education: the NCAAA and the National Framework System. NCAAA works as an independent body for quality assurance for higher education organizations [25]. ETEC launched the Saudi Arabia Qualifications Framework (SAQF) to attain several objectives, such as unifying curriculum designs in educational and training organizations of the higher education sector, identifying necessary learning outcomes for each specialty, and approving academic certificates internally and externally. There are 10 levels in SAQF, which cover all stages and sectors of education and training. These levels describe learners’ outcomes in terms of what the learner is expected to know, understand, and be able to do when graduated. The characterization of these levels can be used as a tool for formulating learning outcomes at different levels to include the complexity of learning outcomes across levels 1 (lowest level of complexity) to 10 (highest level of complexity) [26]. The ETEC underpins all aspects of nursing programs that are conducted by national education bodies.

The Saudi Commission for Health Specialties

The SCFHS is responsible for the professional classification and registration of health specialties in KSA. All health specialties in higher education programs must be approved by the SCFHS. For example, it approves what the necessary knowledge and competencies are for students who graduate from different health programs, and it issues licenses for those who pass in their exams so that they can be employed under the Ministry of Civil Services. However, as part of the national outlook of the Vision 2030, the task of the accreditation of higher education programs has been placed under the authority of NCAAA. Based on the rules of the SCFHS, there are three bodies dominating the nursing profession: 1) the Nursing Department, which is responsible for everyday nursing issues within the SCFHS’s scope; 2) the Nursing Scientific Council, which consists of a nursing expert panel and provides consultation to the SCFHS on nursing education matters, especially postgraduate training programs; and 3) the Council of Professional Nursing Practice that was established by the SCFHS in April 2017, which aims to improve nursing practice, to help to classify health specialty certificates, to create career paths for nurses, to develop solutions for nurses who have failed the Saudi nursing examination, and to develop courses for professional nursing development [27,28].

*SCFHS Postgraduate Professional Programs * 

In response to the national strategic Vision 2030, the SCFHS has taken the lead in providing professional training and preparation programs for nurses. Thirty-eight postgraduate nursing programs were implemented in the 2017-2018 academic year, including the following majors: adult critical care, oncology, cardiology, emergency, neonatal intensive care, and midwifery [29]. In addition to these programs, home care and community nursing, as well as other programs, were established in 2019. These programs are of two years’ duration with a clinical-oriented training and evaluation. The trainee is required to pass a promotion exam to level up in the program and then pass a theoretical exam and the Objective Structured Clinical Examination (OSCE) to be eligible for graduation. A designated scientific committee under the supervision of the SCFHS designs these postgraduate programs’ contents. The method of acceptance into these programs goes through several stages at the SCFHS. Training centers for these programs are healthcare organizations accredited by the SCFHS. Universal training modules, such as patient safety and infection control, are provided through the e-learning platform on the SCFHS website [30].

*Nursing Continuing Education * 

Nurses who need to renew their licenses from the SCFHS are required to complete 15 hours of Nursing Continuing Education (NCE) program for a one-year license, 45 hours for a three-year license, and 75 hours for a five-year license [31]. Continuing education activities include conferences, seminars, workshops, training courses, publishing papers or books, research contributions, accredited online health-related activities, and panel discussions.

Discussion

This review presents a thorough analysis of the evolution of the Saudi nursing education landscape from its beginnings in 1948 until 2020. Despite the scarcity of sources documenting nursing education in KSA, this review has been able to highlight the huge role that governmental agencies and policies have played in the growth and development of nursing higher education in KSA. Furthermore, in contrast to earlier studies that claimed that the first nursing education program had been established by the WHO, this study, by reviewing primary sources, has revealed that the emergence of nursing education programs in Saudi Arabia was indeed led by an American nurse with the support of the Arabian-American Oil Company [6,7,20].

Nursing education in Saudi Arabia has gone through several developmental phases. The first phase started in 1948 and witnessed the emergence of nursing education through the Arabian-American Oil Company initiative. This phase was characterized by male dominance and resulted in the graduation of 256 Saudi and American male nurses to satisfy the Arabian-American Oil Company needs. American initiatives were also evident in the neighboring country of Jordan where American nurses started the MOH School of Nursing in 1953 [32].

The second phase (1958-1975) was driven by the Saudi government's need to increase the production of qualified nurses. The establishment of the first male nursing institute was started by a collaboration with the WHO. The female enrollment into the nursing profession followed shortly in 1961. The third phase (1976-1990) witnessed the involvement of universities in nurses' education either at the graduate or postgraduate levels. In contrast to the first phase, these programs also targeted female students. Male programs did not exist back then. The fourth phase (2004-2020) was the correction phase where male nursing programs opened in several universities at the graduate and postgraduate levels; the phase also saw the establishment of Ph.D. programs. From a nursing professional development viewpoint, the spark was lit by the postgraduate nursing programs by the SCFHS.

Interestingly, exploring the history of nursing education in KSA revealed the exit of MOH as an education provider in 2011 and its reentry as an organizer of training centers for the majority of the postgraduate professional nursing programs in 2017. This may raise concerns about graduates’ educational preparation, taking into account the outcomes of previous changes/variations in the running of the nursing programs. Moreover, hospital-based nursing programs have encouraged private sector hospitals to run these programs as training centers to prepare their nursing staff for specialized nursing care. However, the governance of the SCFHS as a monitoring body may address these concerns. In general, since those postgraduate professional programs have not produced any graduated trainees so far, it is not yet possible to evaluate the outcomes.

Gender-related issues have been evident in Saudi nursing education in terms of students’ academic preparation as well as the estimated needs of healthcare organizations for nursing graduates. Due to cultural issues, male students are not allowed to receive their training in female units, such as the maternity unit and delivery room, which puts an extra burden on nursing schools to provide advanced simulation laboratories to satisfy training needs in these areas. However, there is no evidence to persuade nursing schools to introduce gender quotas for future nursing students. According to an SCFHS report, there are 13,001 female and 4,084 male students currently enrolled in nursing programs [2]. However, the report does not mention the healthcare system’s needs specifically in terms of gender.

One of the most important nursing issues currently is the lack of national nursing scope of practice, which is a part of SCFHS' mandate [6]. This issue works against nursing education development in two ways. Firstly, due to the lack of a national scope of nursing practice, nursing schools are adopting different international scopes, which may lead to variations in learning outcomes. Secondly, there is no guarantee that nursing graduates will practice what they have learned and trained for during their studies. These issues also work against Saudi nurses who graduate from international nursing schools, including advanced nursing practitioners who are not guided by clear nursing practice regulations [33].

Generally, the recent developments in nursing education in KSA in both academic and professional programs are promising. Saudi Vision 2030 may present a great challenge to nursing education due to the need to increase Saudization in the nursing profession by 60% [5]. The accelerated production of graduates by nursing programs and regulating nursing committees will contribute to the growth of nursing as a profession and strengthen the backbone of the healthcare system transformation in KSA. Due to the broadness of the nursing education field and a scarcity of data, the paper did not make an attempt to highlight other issues in the field, such as the challenges faced by the faculty members, simulation proficiency, and availability of clinical training. It is hoped that this paper, by serving as a document that explores nursing education in terms of its history and development in KSA, will at least partly make up for the scarcity of published studies about nursing education in the country.

Recent developments in Saudi nursing education call for academic nursing leaders to participate efficiently in designing nursing strategies and operational plans that can foster the development of national nursing education. Furthermore, the time has come for politicians and planners to participate efficiently in supporting the development of nursing as a mature and independent profession. Lessons learned so far may inspire Saudi nursing leaders as well as nursing bodies in other countries to create strategies to advance the nursing education system. Using the parameters of national healthcare needs and international benchmarks in nursing education will certainly save time and enhance the outcomes of nursing education programs.

## Conclusions

Nursing education in KSA had traditional beginnings similar to those in other countries. However, exploring the historical development of nursing education reveals a picture of steady improvement, especially during the last 15 years. Moreover, the Saudi government Vision 2030 has a significant role in unifying the efforts for the nationwide growth of the nursing field. This calls for academic nursing leaders to participate efficiently in designing nursing strategies and operational plans that can foster the development of national nursing education. Furthermore, It is imperative for politicians and planners to participate efficiently in supporting the development of nursing as a mature and independent profession.
